# Colostrum of Preeclamptic Women Has a High Level of Polyphenols and Better Resistance to Oxidative Stress in Comparison to That of Healthy Women

**DOI:** 10.1155/2019/1380605

**Published:** 2019-02-21

**Authors:** Tali Silberstein, Batel Hamou, Shelly Cervil, Tamar Barak, Ariela Burg, Oshra Saphier

**Affiliations:** ^1^Department of Obstetrics and Gynecology, Soroka Medical Center, Ben-Gurion University of the Negev, Beer-Sheva, Israel; ^2^Department of Chemical Engineering, Sami Shamoon College of Engineering, Beer-Sheva, Israel

## Abstract

Preeclampsia is a common pregnancy complication. Abnormal development of the placenta is the prevailing cause theory of this complication. Women with preeclampsia suffer from acute oxidative stress and high lipid oxidation in plasma. The aim of this study was to compare levels of polyphenols and lipid peroxidation in colostrum of nursing mothers with and without preeclampsia. The study was conducted at the Department of Obstetrics and Gynecology at Soroka University Medical Center. The study group consisting of 18 women, who were diagnosed with preeclampsia, was compared to the control group: 22 healthy women. The total phenolic content in the colostrum was determined by using the Folin–Ciocalteu method. Lipid peroxidation was determined by measuring MDA, using the TBARS assay. Polyphenol concentrations were significantly higher (about 33%) in the colostrum of the study group compared with the control group (*p* = 0.00042). Lipid peroxidation levels (MDA) were significantly lower (about 20%) in the colostrum of the study group compared with the control group (*p* = 0.03). Negative correlation was found between MDA concentration and the polyphenol level (*R* = −0.41, *p* = 0.02). In conclusion, we showed in this study a potential compensation mechanism that protects the newborn of a mother with preeclampsia from the stress process experienced by its mother.

## 1. Background

During pregnancy, there is an increase in the oxidative stress, a process created by a normal systemic inflammatory response; this results in high volumes of circulating reactive oxygen species (ROS). The placenta is the main source of ROS during pregnancy [1–3]. The oxidative stress formed during pregnancy is counteracted by the increased synthesis of antioxidants.

Preeclampsia is a common pregnancy complication that occurs in about 4% of pregnancies. There are two degrees of severity for the preeclampsia syndrome according to the symptoms, physical examination, and laboratory results: mild preeclampsia and severe preeclampsia. The signs and symptoms of preeclampsia are high blood pressure, high concentration of protein in the urine (kidney damage), severe headaches, visual impairment (blurring, temporary vision loss, or strong sensitivity to light), abdominal pain, an increase in the level of liver enzymes indicating liver damage, oliguria and anuria, shortness of breath, laboratory disturbances, preterm labor, and placental abruption. In most cases, some or all symptoms occur after the 20th week [4]. This complication is thought to be abnormal development of the placenta, mostly due to insufficient remodeling of the maternal vasculature perfusing the intervillous space. This may lead to a complex process of ischemia-reperfusion in the placenta with the release of cytotoxic factors into the maternal circulation. The uteroplacental hypoperfusion during preeclampsia increases oxidative stress in both the mother and the fetus [5–8].

The oxidative stress in placental cells is created by free radicals released from the inadequately perfused fetoplacental unit. The plasma membranes of the circulating blood cells can be oxidized when passing through the ischemic placenta contributing to the propagation in this way of the oxidative stress to distal tissues. The antioxidant protection is reduced in preeclampsia, because of a decrease in free radical scavengers and the activity of the antioxidant enzymes [9].

In the biochemical aspect, previous studies have shown that women with preeclampsia suffer from oxidative stress and high lipid oxidation in plasma and lack some antioxidant groups [10].

Polyphenols represent a group of chemical substances that is common in plants, and it is structurally characterized by the presence of one or more phenol units. Polyphenols are the most abundant antioxidants in human diet. The largest and best studied class of polyphenols is flavonoids which include several thousand compounds [11]. The activity of polyphenols as antioxidants is varied: breaking chain reactions involving free radicals, suppressing the formation of free radicals in the cell, and chelating (binding to metallic ions) the free metal ions involved in the creation of radicals [12].

Very few studies have investigated the differences in the composition of colostrum of healthy women, compared with women who suffered from preeclampsia. One research showed that milk LCPUFAs and neurotrophins are altered in preeclampsia. They suggested that LCPUFA could plausibly influence the growth especially in children born to mothers with preeclampsia [13]. It was also shown that in preeclampsia, high cytokine levels in breast milk persist at least for 30 days. These results suggest that preeclampsia may affect milk cytokine balance and offer an immunological signal for the host defense in high-risk neonates [14]. Despite the great importance of polyphenols in the diet, there have been no studies that measured the content of polyphenols in colostrum. In addition, even though polyphenols have potential pathophysiological significance in preeclampsia, we could not find a study that measured their levels in colostrum of women who suffered from preeclampsia in comparison to healthy women colostrum.

The aim of this study was to compare levels of polyphenols and lipid peroxidation in colostrum of nursing mothers with and without preeclampsia.

## 2. Materials and Methods

### 2.1. Sample Collection for Research

Colostrum collection was performed at the Soroka University Hospital in the Department of Obstetrics. A total of 40 nursing women participated in the study and were divided into two groups: the control group consisted of healthy pregnant women (*n* = 22) and the study group consisted of pregnant women who were diagnosed with mild (4) or severe preeclampsia (14) (total *n* = 18). However, the mild preeclamptic women were almost severe, and we decided to analyze them with the severe group. Women with preeclampsia usually stay after delivery in the delivery room for close follow-up and treatment. Usually, their situation is not easy, and due to that and the fact that they want their milk for the baby, it is not easy to recruit them for the study. The sample sizes were decided to be around 20, a number that can allow us to do good statistical analysis and is achievable. Since in our hospital around 50% of the parturient are Bedouins and 50% are Jews, we are very familiar with the Arabic language and with this population. Recruitment of the women to the study was done before they gave birth. After having discussion with the women, the following procedures were performed. After washing hands and cleaning the nipple, the women pressed and squeezed the nipple for colostrum. This was done in the presence and guidance of the physician that recruited the woman. Primary milk, colostrum, was collected during the period of day one to day 7 after delivery. Milk samples were kept in the refrigerator at a temperature of −20°C until assays were performed. The collection of samples for research purposes was approved by the Ethics Committee of Soroka Hospital, in accordance with the Helsinki declaration. All the women recruited for the study signed on a document of informed consent. The women were asked to characterize their diet (vegetarian vs. regular) and whether they smoke cigarettes or consume alcohol.

### 2.2. Determination of Total Polyphenol Content in Colostrum

The total phenolic content in the colostrum was determined by using the Folin–Ciocalteu method [15]. This method is based on the redox reaction of the reagent forming a blue color pigment with typical absorbance at 760 nm. All UV-Vis measurements were performed using a Cary 100 Bio, UV-Visible spectrophotometer.

### 2.3. Extraction of Polyphenols from Colostrum

In order to extract the polyphenol from the colostrum, we developed optimum condition for extraction. The goal was to extract maximum polyphenols, while at the same time receiving a clear solution without proteins. 1.0 ml of colostrum was added to a 10 ml polypropylene centrifuge tub, followed by adding 1.0 ml of pure water, 2.0 ml of pure ethanol, 0.6 ml of TCA 20% (three chloroacetic acid), and 0.4 ml of 1.0 M HCl (HCl was added in order to prevent oxidation of polyphenols during extraction and assay processes). The mixture was stirred in a vortex for several seconds and was bathed in the bath for 20 minutes. The mixture then underwent a 20-minute centrifugation for 3500 g (Heraeus Labofuge 200 centrifuge), with the aim of depositing all the solids and receiving a clear solution.

An aliquot of 0.5 ml from the extract fluid was mixed with 1.25 ml of the Folin–Ciocalteu reagent (FCR) and 2.0 ml saturated solution of sodium carbonate. This solution was vortexed for 15 seconds and was kept in the dark for 30 minutes for color development. Then, the solution was centrifuged (5300 rpm) for 10 minutes until it was transparent. The supernatant was collected, and the solution absorbance was measured by using a UV-Vis spectrophotometer (Jasco V-730 Spectrophotometer) at a wavelength of 760 nm. The total amount of polyphenols was expressed as gallic acid equivalents (GAE), based on the calibration curve.

### 2.4. Lipid Peroxidation Measurement

**Figure 1:**
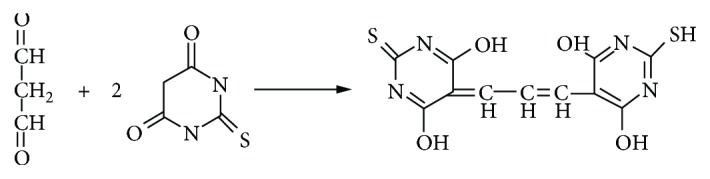
Polyphenol levels in colostrum of healthy and preeclamptic women. The results are shown in mM equivalents of gallic acid. Each column represents mean ± standard deviation.

**Figure 2:**
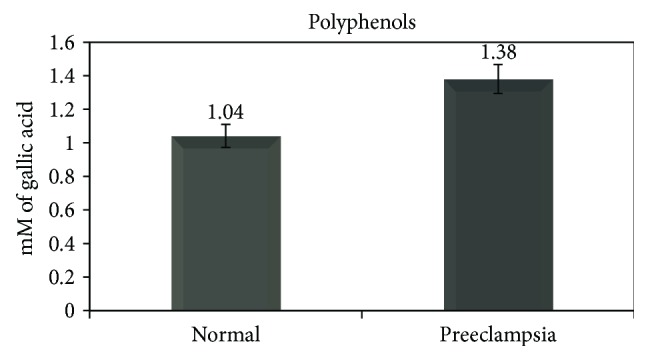
A chemical reaction between MDA and TBA to yield a pink chromogen [9].

Lipid peroxidation was determined by measuring the oxidation product of lipids, malondialdehyde (MDA). This molecule is well known as a biomarker and serves as a reliable indirect measure of lipid oxidation. MDA levels were measured spectroscopically by the thiobarbituric acid reactive substances (TBARS), according to a procedure already published by Halliwell and Gutteridge [15] with minor modifications. The assay was based on the thiobarbituric acid (TBA) reaction, a reaction between oxidized lipids and solution of 2-thiobarbituric acid under acidic conditions to yield a pink chromogen with a maximum absorbance at 532 nm (Figure 1) [15]. An aliquot of 1.0 ml of the colostrum slurry (1 ml) was transferred to a 15 ml tube followed by successive additions of 0.67% TBA in 20% TCA and 0.8% BHT in ethanol. The mixture was then homogenized and centrifuged at 5300 rpm for 10 min and incubated in a 70°C water bath for 20 min. Samples were subsequently cooled under tap water for 5 min and centrifuged for 10 min to separate flocculent material. The color produced by the chemical reaction was read at 532 nm against a blank reaction mixture, and the amount of MDA formed was determined by using the molar extinction coefficient *ε*(530 nm) = 1.56 × 10^−5^ cm nmol^−1^ [16, 17]. Two assays were carried out for the samples: the Folin–Ciocalteu assay for total polyphenols and the TBARS assay for lipid oxidation measured by determining MDA levels (Figure 2). All results are average of three repetitions.

### 2.5. Statistical Analysis

All statistical analyses were performed using the SPSS 11.0 program for Windows. Values of variables were expressed as mean ± SD.

## 3. Results and Discussion

Table 1 presents the characteristics of the study and control group women. In the study group, 14 women were diagnosed with severe preeclampsia and 4 women with mild preeclampsia. Thirteen women were treated with magnesium sulfate.

Polyphenol concentrations are shown in Figure 1. Polyphenol concentrations were significantly higher (about 33% higher, *p* = 0.00042) in colostrum of preeclamptic women in comparison to healthy women. This result surprised us, given the fact that we expected the opposite results. Preeclampsia is known to be a clinical syndrome with a mechanism of oxidative stress. Therefore, it was likely that polyphenol levels would be higher in healthy women's colostrum than in preeclamptic women's colostrum. In the study group, no difference was found in polyphenol levels between women treated and not treated with magnesium sulfate.

**Figure 3:**
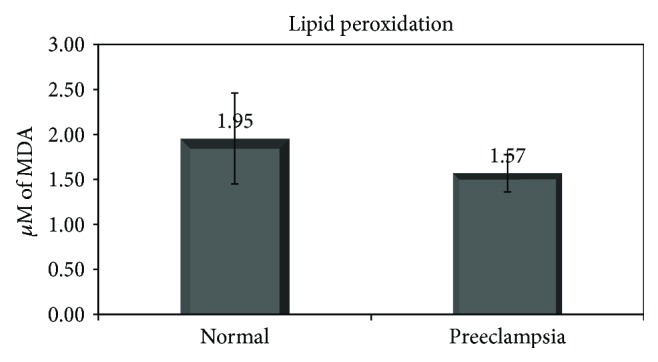
Levels of lipid oxidation in the colostrum of healthy and preeclamptic women. Results are shown in *μ*M of MDA. Results are shown as mean ± standard deviation.

Lipid peroxidation levels are shown in Figure 3. The results are shown as the concentration of MDA, which is a measure of lipid oxidation. Significantly lower levels of lipid oxidation were found in the colostrum of women with preeclampsia compared with healthy women (about 20%, *p* = 0.03). In the study group, no difference was found in polyphenol levels between women treated and not treated with magnesium sulfate.

Again, this result surprised us; however, it works well with the fact that levels of polyphenols, which act as antioxidants, were higher in the colostrum of the preeclamptic population. We assumed that high levels of polyphenols increase the resistance of the milk to the oxidative stress, so there is a reduction in the level of lipid oxidation.

**Figure 4:**
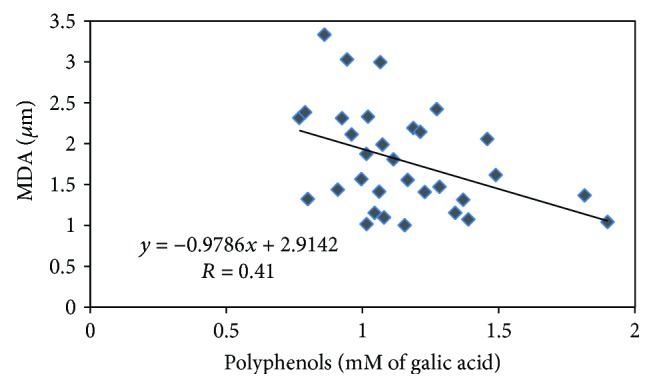
Pearson correlation between the level of lipid oxidation (MDA) and polyphenols in colostrum of healthy women and women with preeclampsia.

The results indicated higher levels of polyphenols in the colostrum of women that suffers from preeclampsia and lower levels of lipid oxidation. These results appear to be in contradiction to the fact that the mechanism of preeclampsia involves a state of oxidative stress originating from the placenta. At the same time, the explanation for the special phenomenon may be derived from the compensation mechanism in the mother. During the onset of the disease, breast milk may be enriched with polyphenols more in preeclamptic women than in healthy women. This seems to have an evolutionary advantage in protecting the fetus exposed to oxidative damage during labor. The fetus that has suffered from oxidative stress at birth can overcome the damage or prevent further damage by immediate diet of colostrum rich in polyphenols. In order to show whether lipid oxidation levels were correlated with colostrum and polyphenol levels, we combined all the results of the study into one group and examined whether there was an inverse correlation between MDA and polyphenol levels in colostrum. We therefore drew a scatter graph of MDA levels depending on the levels of polyphenols in colostrum. The Pearson correlation coefficient was determined for the regression line and the significance level of the adjustment coefficient. The results are shown in Figure 4.

A Pearson correlation of -0.41 was obtained between the level of lipid oxidation and polyphenols in colostrum with significance (*p* = 0.02). This means that the higher the levels of polyphenols, the lower the MDA levels. The value of the Pearson coefficient, -0.41, indicates that at a 98% significance level, polyphenols contribute about 40% to the prevention of oxidation of lipids in colostrum. It is likely that other factors, such as activity of antioxidant enzymes and genetic factors, also are involved and influence the process. We analyzed the results according to the ethnicities. No differences were found between the ethnicities. In the preeclampsia group, 11 had CS compared with 5 in the control group. When we analyzed the results within the groups with/without CS, we found that in the control group, a higher level (13%) of MDA was measured in the CS delivery compared with vaginal birth, and a higher level of polyphenols in the vaginal birth compared with CS. No difference was found in the preeclampsia group between those with and without CS. These results support our thesis even more although statistical significance was not reached. Only minority of women had epidural anesthesia: one in the control group and 3 in the preeclampsia group. This did not affect the results.

## 4. Conclusions

This work has interesting and important conclusions. The colostrum of women with preeclampsia was found to be richer in polyphenols than that colostrum of healthy women. Levels of lipid oxidation were found to be lower in the preeclampsia group. High levels of colostrum polyphenols appear to affect the decrease in lipid oxidation. All differences are statistically significant. The main conclusion is that the woman's body apparently has a compensation mechanism that protects the newborn from the stress processes the mother experiences. These results are consistent with studies showing an increase in the levels of components in breast milk that are supposed to provide protection for the baby in the group of women with preeclampsia [13, 14]. It was shown that milk LCPUFAs and neurotrophins are altered in preeclampsia. LCPUFA could plausibly influence the growth especially in children born to mothers with preeclampsia (18). Preeclampsia may affect milk cytokine balance and offer an immunological signal for the host defense in high-risk neonates [14].

Our recommendation following this study is to encourage women who have experienced preeclampsia to breastfeed their children in the first days of life. We believe this is very important in protecting their newborn.

Our study has potential limitations mainly due to its sample size. In addition, there are more methods to measure polyphenols. We think that similar results to those of other/different methods could support our study results.

## Figures and Tables

**Table 1:** Characteristics of women.

	Study group (*n* = 18)	Control group (*n* = 22)
Age	29 ± 7	30 ± 6
Regular nutrition	22	22
Gestational age (w)	36 ± 4	38 ± 2
Cigarette smoking	None	None
Magnesium sulfate treatment	13 (72%)	None
Spontaneous vaginal delivery	4 (22%)	16 (78%)
Cesarean section	14 (73%)	6 (27%)
Colostrum age (d)	5 ± 1	4 ± 1

## Data Availability

The data used to support the findings of this study are available from the corresponding author upon request.
